# Undertaking multi-centre randomised controlled trials in primary care: learnings and recommendations from the PULsE-AI trial researchers

**DOI:** 10.1186/s12875-023-02246-8

**Published:** 2024-01-02

**Authors:** Kevin G. Pollock, Carissa Dickerson, Manjit Kainth, Sarah Lawton, Michael Hurst, Daniel M. Sugrue, Chris Arden, D. Wyn Davies, Anne-Céline Martin, Belinda Sandler, Jason Gordon, Usman Farooqui, David Clifton, Christian Mallen, Jennifer Rogers, Nathan R. Hill, A. John Camm, Alexander T. Cohen

**Affiliations:** 1 Bristol Myers Squibb Pharmaceutical Ltd, Uxbridge, UK; 2 Health Economics and Outcomes Research Ltd, Cardiff, UK; 3 Primrose Lane Health Centre, Wolverhampton, UK; 4 School of Medicine, Keele University, Staffordshire, UK; 5 University Hospital Southampton, Southampton, UK; 6 London Heart Practice, London, UK; 7 Service de Cardiologie, Université de Paris, Innovative Therapies in Haemostasis, INSERM, Hôpital Européen Georges Pompidou, 20 rue Leblanc, Paris, France; 8 Institute of Biomedical Engineering, Department of Engineering Science, University of Oxford, Oxford, UK; 9 PHASTAR, London, UK; 10 Cardiology Clinical Academic Group, Molecular & Clinical Sciences Research Institute, St. George’s University of London, London, UK; 11 Department of Haematological Medicine, Guys and St Thomas’ NHS Foundation Trust, King’s College London, London, UK

**Keywords:** Qualitative research, Reflection exercise, Randomised controlled trial, Primary care, Recruitment

## Abstract

**Background:**

Conducting effective and translational research can be challenging and few trials undertake formal reflection exercises and disseminate learnings from them. Following completion of our multicentre randomised controlled trial, which was impacted by the COVID-19 pandemic, we sought to reflect on our experiences and share our thoughts on challenges, lessons learned, and recommendations for researchers undertaking or considering research in primary care.

**Methods:**

Researchers involved in the Prediction of Undiagnosed atriaL fibrillation using a machinE learning AlgorIthm (PULsE-AI) trial, conducted in England from June 2019 to February 2021 were invited to participate in a qualitative reflection exercise. Members of the Trial Steering Committee (TSC) were invited to attend a semi-structured focus group session, Principal Investigators and their research teams at practices involved in the trial were invited to participate in a semi-structured interview. Following transcription, reflexive thematic analysis was undertaken based on pre-specified themes of recruitment, challenges, lessons learned, and recommendations that formed the structure of the focus group/interview sessions, whilst also allowing the exploration of new themes that emerged from the data.

**Results:**

Eight of 14 members of the TSC, and one of six practices involved in the trial participated in the reflection exercise. Recruitment was highlighted as a major challenge encountered by trial researchers, even prior to disruption due to the COVID-19 pandemic. Researchers also commented on themes such as the need to consider incentivisation, and challenges associated with using technology in trials, especially in older age groups.

**Conclusions:**

Undertaking a formal reflection exercise following the completion of the PULsE-AI trial enabled us to review experiences encountered whilst undertaking a prospective randomised trial in primary care. In sharing our learnings, we hope to support other clinicians undertaking research in primary care to ensure that future trials are of optimal value for furthering knowledge, streamlining pathways, and benefitting patients.

**Supplementary Information:**

The online version contains supplementary material available at 10.1186/s12875-023-02246-8.

## Introduction

Research is essential to further our understanding of healthcare and medicine. However, conducting effective and translational research can be challenging for researchers, with difficulties in recruitment, inequalities or bias in representation, and operational issues presenting barriers to effective research. Whilst the UK is an international leader of researcher in primary care, [1] engagement amongst trainees in general practice to undertake research is limited [2]. The UK’s National Institute for Health and Care Research (NIHR) is championing for research to be embedded within health and social care, [3] however it is important to understand the challenges and barriers to conducting research to ensure researchers are supported in conducting research that is of value to patients, clinicians, and the wider healthcare system.

Recruitment is a well-recognised challenge within randomised controlled trials, [4, 5] with many trials failing to meet recruitment expectations [6–9]. Recruitment to trials in primary care settings face additional challenges as many consultations are now via telephone or online as a result of the COVID-19 pandemic and patients are less intensively managed than in secondary care; both features resulting in practice researchers having fewer opportunities to facilitate recruitment in potential participants. Trials evaluating screening interventions are also likely to encounter recruitment challenges, as otherwise-well patients are invited to undertake an intervention that may result in them being diagnosed with a condition. However, even if recruitment is satisfactory, completion of the study faces ongoing challenges, with patient discontinuation a common barrier [10].

We undertook a multicentre randomised controlled trial in the primary care setting in England. During the trial we encountered numerous challenges relating to recruitment, disruption due to the COVID-19 pandemic, the use of new technology for home-based screening, and general trial operations. Very few trials undertake a formal reflection exercise following the completion of the trial, [11, 12] potentially missing valuable opportunities for the sharing of learnings to optimise trial conduct and outcomes. Therefore, we sought to reflect on our experiences throughout the trial and – given their likely applicability – share our thoughts on these challenges, the lessons learned, and our recommendations for researchers undertaking or considering research in the primary care setting, in the hope that they may be of value for other researchers within primary care.

## Methods

### PULsE-AI trial overview

The Prediction of Undiagnosed atriaL fibrillation using a machinE learning AlgorIthm (PULsE-AI) trial was a prospective, multicentre, randomised, controlled trial conducted in England from June 2019 to February 2021 [13, 14]. In short, 23,745 participants who met eligibility criteria were identified from medical records at participating practices. All eligible patients were individually randomised into intervention or control arms. Following randomisation, a risk prediction algorithm – developed using machine learning techniques – was applied to medical records of all eligible participants to generate an individualised score for the risk of undiagnosed atrial fibrillation (AF). Participants randomised to the intervention arm and identified as high risk of undiagnosed AF by the algorithm were invited to attend the research clinic for diagnostic testing. Participants who accepted the invitation underwent a 12-lead electrocardiogram (ECG) and – if their 12-lead ECG was negative and they had access to a compatible smartphone or tablet – two-weeks of home-based ECG monitoring using a KardiaMobile portable ECG monitor. Patients diagnosed with AF (or other arrhythmia) as a result of the trial were referred for further care as per routine practice. Participants in the intervention arm at low risk of undiagnosed AF and all control arm participants were managed routinely and had no contact with investigators. The trial involved an industry sponsor, a Contract Research Organisation, academic Clinical Trials Unit, the National Institute for Health and Care Research Clinical Research Network (CRN), and six practices across the West Midlands in England. In addition, the trial was impacted by the COVID-19 pandemic, and was paused from 16 March 2020 to 21 July 2020.

### Participants

All researchers involved in the PULsE-AI trial were invited to participate in the reflection exercise. Invited researchers included members of the core trial team/Trial Steering Committee (TSC) (including trial logistics personnel, clinical experts, scientific advisors, and industry sponsors) and general practice-based Principal Investigators (PIs) and their research teams. Practices were reimbursed financially for their time to undertake the reflection exercise.

### Reflection exercise

Approximately 17 months following completion of the PULsE-AI trial, eligible participants were invited to participate in the qualitative reflection exercise. Members of the TSC were invited to attend a semi-structured focus group session, and PIs and their research teams were invited to attend a semi-structured interview. This mixed approach was taken because by working together throughout the trial the TSC was closely aligned, whereas PIs and their research teams at individual practices had different experiences of the trial. The focus group and interview sessions were led by one of the trial researchers; this approach enabled the interviewer to probe for additional detail where required, but their opinions were not captured as part of the exercise. The focus group and interview questions are included in Supplementary File S1. All participants provided verbal informed consent prior to participating in the interview/focus group sessions for this reflection exercise. All focus group and interview sessions were undertaken virtually via Microsoft Teams® and recorded and transcribed using the platform’s inbuilt software. All transcriptions were checked for accuracy and edited where required. Ethical approval was granted by the Wales Research Ethics Committee 5 and the study was approved by the Health Research Authority (HRA) and Health and Care Research Wales (HCRW) (IRAS project ID: 252,934).

### Analysis

**Fig. 1:**
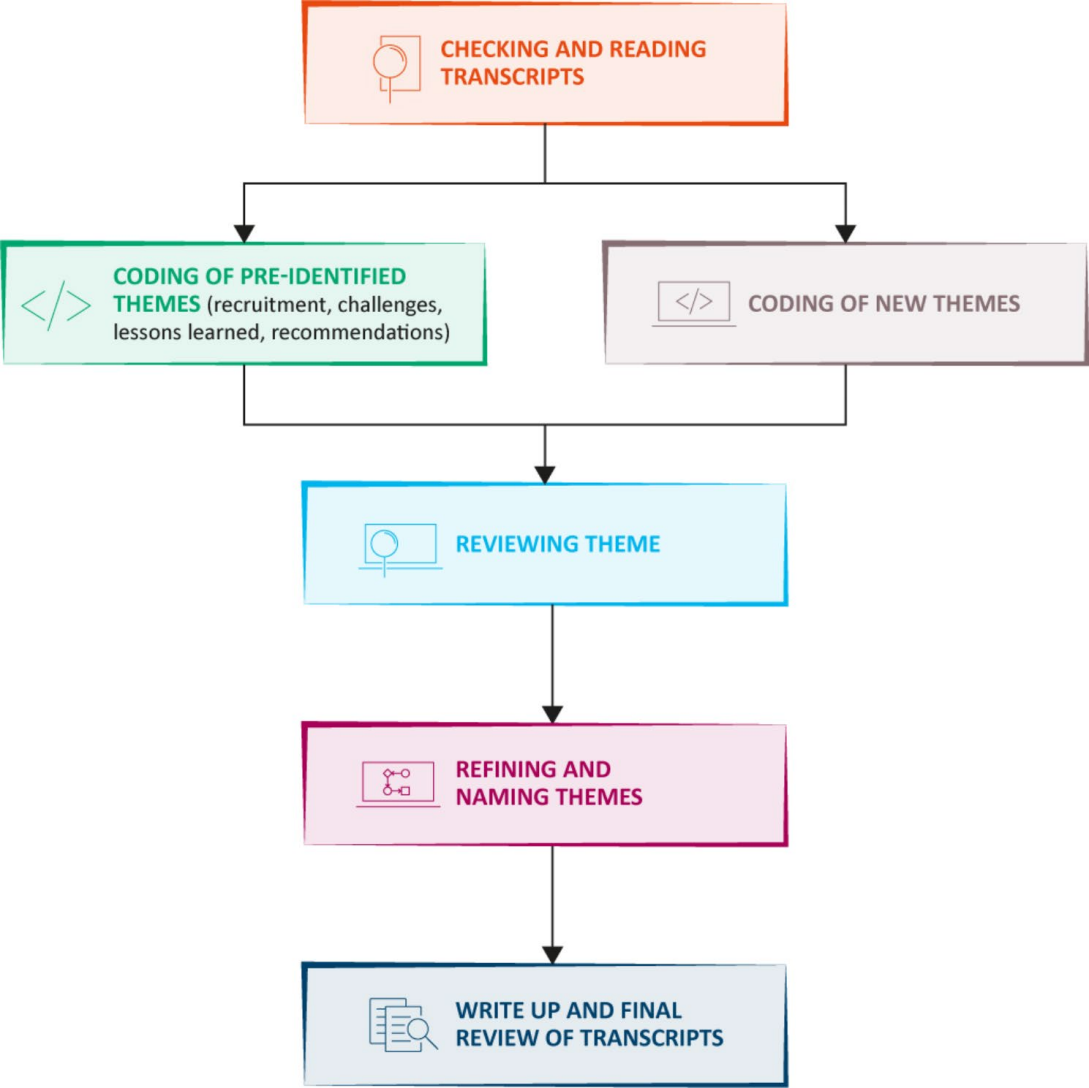
Overview of the thematic analysis process

Thematic analysis was undertaken following the methods outlined by Braun and Clarke [15]. Some themes were pre-identified to form the structure of the focus group/interview sessions (recruitment, challenges, lessons learned, and recommendations) and others emerged during analysis based on a reflexive approach [16]. Following transcription, pre-identified and new themes were identified, colour coded and transferred into a Microsoft Excel® spreadsheet to enable organisation and filtering of responses for interpretation. Common responses to each theme were grouped and relevant supporting quotations identified for reporting. Themes were reviewed, refined where appropriate, and named. Analysis was undertaken by a researcher independent to the trial, and checked by a second researcher (who led the focus group and interview sessions and who was involved in the trial). All transcripts were reviewed for a second time by the senior researcher alongside the drafting of the manuscript to ensure all relevant information was captured and no important themes were left unidentified. This process is outlined in Fig. 1. Researchers were also asked questions relevant to the PULsE-AI trial regarding the use of artificial/augmented intelligence (AI) in healthcare and COVID-19. However, these have not been included in detail in this manuscript to ensure our recommendations are as generalisable as possible to all interventional research within primary care.

## Results

Of the 14 members of the TSC, seven attended one of two focus group sessions, and one member answered the questions in writing. The eight members of the TSC who participated included, three members of the trial project team from the industry sponsor, two researchers from the academic clinical trials unit, twotrial cardiologists, and the Chief Investigator. Of the six general practices involved in the PULsE-AI trial, one accepted the invitation to participate in the reflection exercise. Of the nine researchers who participated in the reflection exercise, eight were male.

### Recruitment

#### How response rates compared with expectations

Across all six practices involved in the PULsE-AI trial, 28% (n = 255) of patients invited to participate in the trial accepted the invitation. Of the eight members of the TSC who participated in the research, five (63%) felt that response rates were similar to what they expected, two (25%) felt they were higher than expected, and only one (13%) reported that response rates were lower than they had expected. The one PI who participated in the research felt that response rates for his practice were lower than expected.

For me, the percentage of 28% was actually surprising, I thought it would be lower! … Given the pandemic, and all that went with it, … 28% is probably a success rather than a failure.

I did think it was lower than expected, …So I spent around five years working in primary care, running observational research studies, … and for the age group we were targeting [over 65s] … we’d normally get around 40% response rate … maybe I was being unrealistic, but I would have expected 40[%] ish.

#### Recommendations to improve response rates

**Fig. 2:**
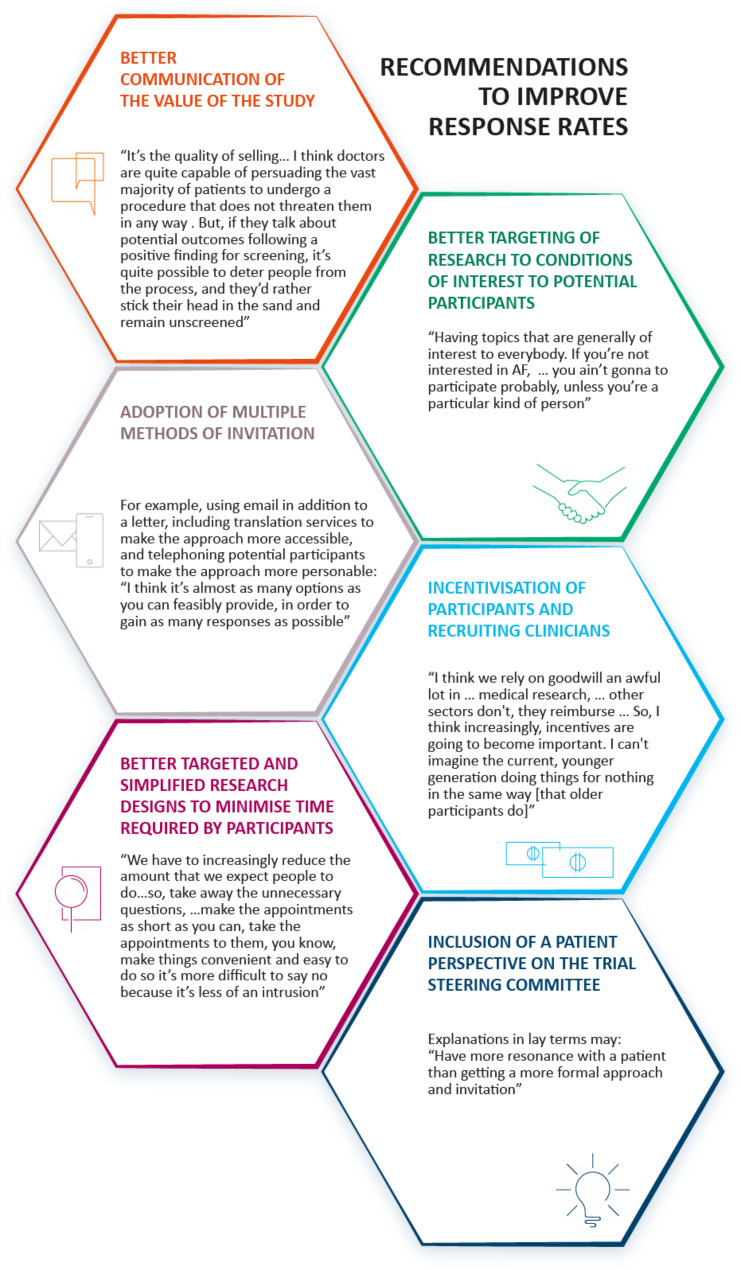
Summary of recommendations to improve participant response rates to trials in primary care

As a result of the reflection exercise, there were a number of recommendations for suggested ways to improve response rates in primary care trials (Fig. 2).

### Challenges

#### Key challenges encountered by practices

Recruitment and technology were the key challenges encountered by the practice interviewed:

The only real challenges we had were… A, the recruitment, so just, you know, prompting and getting people in, and B, the equipment, the technical equipment [the KardiaMobile device for home-based ECG recording].

At the start, there’s quite a bit of inertia, you know, it can be quite daunting to, to get the computer kit working.

The burden of trial logistics and operations were also stated as a challenge. Whilst the practice interviewed was relatively small, they commented that larger practices with higher numbers of eligible patients may have struggled more with this aspect of involvement and highlighted the potential role the CRN can play in supporting involvement in research:

The biggest thing I’d like to say is … you need a team around you … if you’re in a team and you can’t build a team around you, … that’s where the, the CRN nurses become helpful, because I think having them helping with research, A, it creates a team and I think B, it can help drag other members of the clinical team along and into, into research.

#### Key challenges encountered by the TSC

A lack of clarity around roles and responsibilities was highlighted as one challenge by members of the TSC. This was predominantly related to the fact that whilst the CRN did provide some support for this trial, because it was industry-sponsored, their remit was reduced (compared with a non-industrial trial), ultimately meaning the practices had additional responsibilities. Overall, it was:

“Difficult to know where responsibilities started and stopped, and I think particularly since the CRN are used to working in primary care, in non-industrial trials, whereas actually the responsibilities of the practice are much greater for the industrial studies than it is for a traditional portfolio study. A lot of stuff ended up being in no man’s land in terms of activity and who should be doing it and when, and I think that caused confusion,…having that really hammered out at the very start would have been, would be very useful moving forward.

Practice engagement was also stated as a challenge in some instances, especially in busy practices. Whilst some practices appreciated the communication, others did not, but COVID-19 also likely played a role here:

I think some practices, whilst they appreciated it, it irritated other practices … one model doesn’t fit all.

COVID …I think did have a big impact on the trial. Anything not COVID-related wasn’t prioritised by government demand, so that then makes it very difficult to have to keep engaging, re-engaging, stopping, starting, when there’s an awful lot of other stuff going on Research is never gonna be a priority, or not, not priority, It’s never gonna be the first priority of the practice. That’s patient care. When you’re having to stop, start, stop, start, that does create an, an, awful lot of barriers to it.

The more technical aspects of the trial also provided a challenge. Notably the ‘search and report’ function that practices had to run within their electronic records systems to identify eligible patients:

The technical aspects at the beginning were actually very complex, to extract the data, …it would have been great if we could have somebody go in and run those searches for them at the centres, because they were highly complex extraction techniques to find the population on which we wanted to run the algorithm…having somebody go in makes it a lot easier than doing a phone consult to help them do it.

Also, the use of the KardiaMobile devices for home-based ECG monitoring. These devices required a smartphone and App in order to record the ECG. Smartphone access and/or technological confidence was a barrier:

“The mobile phones were our biggest barrier, and, and, it’s, it’s unfortunate because that was an exclusion criteria for a large proportion, of, of GPs. So having a single-lead ECG that wouldn’t need a phone would have been preferable, and such things exist now, but unfortunately they didn’t then”.

### General recommendations

The main recommendations suggested by participants in this reflection exercise were based on the following themes:

Better communication between parties, including more face-to-face support for practices from the core trial team, and therefore better development of rapport/relationships:

I feel like I had more engagement with those practices where I went to the …site initiation visit, because you knew who you were talking to, you could picture their face, you could picture their setup, …that, sort of, helped those relationships and conversations. … I wish we’d … spent more time with our practice links, understanding how they updated their systems, recorded things, because there was a lot of to-ing and fro-ing … and we needed to find out all of these, like, missing parts of the jigsaw around practice operationalization, … that was integral …to track the progress of the practices and results.

Clarity around roles and responsibilities, particularly when there are multiple parties involved in the trial:

A clear delegation of responsibilities right from the start might have been one way to facilitate that, the expectations around who’s doing what, where, and when sort of thing.

Better utilisation of online-based tools for the sharing of information such as trial enrolment/completion logs:

If we were doing it again, we’d use a lot more online methodologies, and, you know, we’d have shared tools for completion, rather than this sheet we were emailing each other back and forth. Technology has moved on to enable, we’d hope, more efficient processes if we were to do it again.

Improved guidance and support for new methods/technologies. Technology support was one of the key challenges encountered by the practices, therefore methods to better support researchers are a key recommendation:

The hardest bit I found was the technical side, getting the …device set up, getting them reading [ECGs], … when we had the site initiation visit someone did go through it with us, it, was just perhaps um, you know, you could have a video, a YouTube video, or you know, some more pointers or advice, because people do forget.

Incentivisation for practices (to ensure there is someone with primary responsibility within the practice). At the restart of the trial (July 2020) we implemented a ‘Study Champion’ contract with each practice with a £1,000 payment attached:

Once we implemented the practice champion for the study, that really helped things as well, that was incentivised… because there was a payment that came with that. But it did put somebody, a named person responsible that we could fall back on, and then the practice could lean on in order to answer our questions … so maybe including that a little bit earlier in such a study again, would be good.

But also, incentivisation and/or reimbursement for participants to ensure they aren’t ‘out of pocket’, doing so ensures the research is:

“Conducted equitably, in terms of, rural and social factors, and deprivation, you know, in terms of inequality … that may be difficult to do, but there should be due attention given to that collection of data [ECGs], where it as equitable as possible, in terms of access to primary care.”

### Lessons learned

#### General

Given that study duration can impact the outcome of the trial, it is important to implement the intervention quickly. Getting processes set up ahead of ‘pushing go’ is essential to ensure a time-effective trial:

Doing all of that up front, and getting as much information as possible, early, assists the running of such a study.

More consideration for outcomes early on:

“Doesn’t matter how hard you think about stuff, there’s always stuff you miss. You can’t anticipate everything ahead of time… [which is] why we need to be adaptable in our approach to trials, but unfortunately, we work within a system where it takes a long time to do an amendment. But it is what it is, you can’t anticipate everything, and, when it becomes apparent, hopefully it’s not too late to do something about.”

It’s the old adage isn’t it, …measure three times, cut once. It’s probably the same for trials, right? Think it through three times, do it once.

Whilst there is value in having a large amount of expertise involved in the trial, it can also create additional challenges:

Having the amount of expertise there was great, but also everybody had different opinions on lots of things, and so decision making could take a bit more time than usual, and it was all a little bit more complicated … sometimes bigger isn’t better, and smaller, and simpler actually can be a lot more straightforward.

#### Primary care-specific

The value of trials, especially in primary care:

I think patients find it helpful, and that’s a big clinical lesson that we’ve learned, you know, trials are useful. Um, people may think that they’re extra work and, you know, distract from normal work and create extra workload, but I think they’re useful and they help. It’s a good way of helping our patients, you know… we picked up, obviously there would have been diagnoses that we picked up, and, you know, ECG abnormalities that we picked up that needed cardiological opinion. So that was helpful. So, I think we helped our patients.

One model does not fit all.

When you work with hospitals, the whole of the hospital does things in a similar way, but when you work with five practices, they’ll do things five different ways… So, I think, I think we could have spent more time getting to know the individual practices, and finding out how they work and how things would work for them.

#### COVID-19

One of the key lessons learned was related to time and trial duration. The ‘stay at home’ message from the UK government in response to the COVID-19 pandemic was announced in March 2020, nine months into the trial. As a result, the trial was paused for four months prior to being restarted. During this time, it was not possible to actively screen for AF. However, background cases of AF in both intervention and control arms were diagnosed:

I just would have loved to have done it quickly, you know, and, and, because the lag time kills you … If we get interrupted like that again, we should have two cohorts. We should… just try and finish one off, and not drag it out for six months…, or nine months, while you’re waiting for things. Just do, do a bit, and then come back to it when things settle down.

## Discussion

Whilst researchers involved in the PULsE-AI trial encountered numerous challenges during the study, overall feedback relating to involvement was positive amongst those surveyed. Some researchers had prior experience of RCTs in primary care, whereas many were new to research in this setting. Given qualitative evaluations of researcher experiences of RCTs are relatively uncommon, [12] we sought to undertake a reflection exercise to collate perspectives from researchers involved in the trial and disseminate our learnings with other researchers undertaking, or considering research in primary care settings. Key themes that emerged from the reflection exercise included recruitment, incentivisation, and trial operations.

### Recruitment

On reflection, the majority of researchers felt that the response rates (of 28%) were similar to what they would have expected for a trial in primary care. Similar trials in primary care settings have reported response rates ranging from 8–33%, [17–20] which generally accord with what we observed. However, at the beginning of the trial, initial response rates were relatively low, resulting in somewhat unexpected recruitment challenges. Consequently, we added additional interventions, such as a phone call to follow up on recruitment letters and a ‘pop up’ within the medical records of patients randomised to the intervention arm and identified as high risk of undiagnosed AF to prompt a conversation about the study during a consultation, in an attempt to facilitate recruitment. These interventions appeared to result in a small uplift in participants consenting, and analysis from a recent Cochrane Review on strategies to improve recruitment indicates this uplift can be in the region of 6% [5].

The quality of communication of the value of participating in a study is also an important consideration for maximising response rates. The exact methods will likely depend on the trial population of interest; online-based methodologies such as email campaigns and social media are likely to be more effective for younger participants, whereas older generations are likely to benefit from a mixed approach to recruitment to reflect variations in technology use amongst this cohort. Irrespective of method of recruitment utilised, ensuring approaches are targeted and materials are accessible to the cohort of interest may help improve response rates. Furthermore, ensuring trials are accessible, equitable, and inclusive is also essential to ensure that study populations are more reflective of patient populations and therefore trial outcomes are more widely applicable [21, 22]. The use of recruitment approaches that are more likely to resonate with minority groups are required to encourage greater equality and reduced bias in trial participation.

In the PULsE-AI trial, the smallest practice had the highest response rate to invitation. Good clinician-patient relationships are important to this practice and potentially highlight an important link between relationships and the ability of practice teams to encourage participation in trials in this setting. Overall, no one strategy emerged that, if implemented, will have a impact on response rates, but likely an amalgamation of multiple small changes or interventions that, when combined, will potentially maximise participant responses.

### Incentivisation

Incentivisation and reimbursement of time and/or expenses will likely play a greater role in research in the future. Our researchers commented that we rely on goodwill a lot in medical research and they believe that younger generations in the future are unlikely to participate in medical research at the same rate as older generations today, a trend also observed in blood donation [23]. However, there are important ethical factors to consider when offering incentives, particularly that the incentive may reduce an individual’s perception of the risk of participating in the trial, therefore preventing them from providing fully informed consent [24, 25]. Whether incentivisation actually increases recruitment is not well understood; a study reporting on two trials found incentivisation only boosted recruitment in one of the two trials [26]. Ultimately, incentivisation is a subject that could be considered during protocol design, especially in trials where researchers foresee challenges with recruitment. Reimbursement for travel expenses incurred may also support the move to ensure research is conducted equitably and potential participants in more socially deprived and/or rural areas are not dissuaded from participation due to financial or logistical limitations.

### The use of home-based ECG monitoring technology

One of the key challenges encountered by researchers in the trial was the technology. The KardiaMobile device used for home-based ECG monitoring requires a compatible smartphone or tablet to record data. Although newer technologies are now available that remove the need for a compatible smart device, alternative options were unavailable at the time. The mean age of participants in the trial identified as at high risk of undiagnosed AF was 78 years, and consequently, many participants did not have access to or lacked the technological confidence to use a smartphone/tablet. There were also additional complexities (beyond standard use of the device) relating to the need for trial data to be uploaded to secure servers for later cardiologist review. The demographics of the trial population combined with these additional complexities placed substantial burden on researchers, who would have appreciated greater support (e.g. video demonstration in addition to written instructions) in managing this component of the trial. As researchers, it is important to remember that practices involved in research are usually doing so in addition to their normal clinical workload, and therefore any support that can be provided to make learning and implementation of new processes simpler may facilitate greater practice engagement with the trial. Furthermore, a pilot study could be undertaken to try and identify any potential issues – such as the issue we encountered with a number of participants not having access to a compatible smartphone/tablet – early and allow time to explore alternative options if suitable.

### Trial operations

Given the importance of the time element in our trial, and the need to implement the intervention as quickly as possible to detect undiagnosed AF, if we were to undertake the trial again, we would ensure all practices are set up and ready to go concurrently. Whilst this may be impractical for larger trials with multiple centres, where possible, we recommend ensuring all participating practices are identified and contracted with, well ahead of undertaking site initiation visits. Once all practices are ready to begin, the trial can be initiated, and early weeks are not ‘lost’ to these set-up activities. Whilst it is impossible to plan for all eventualities, when designing trials where time can impact the primary outcome, there may be value in upfront planning where possible in how to manage potential challenges such as slow recruitment.

Communication is also a key component of trial operational success. Where possible, face-to-face contact between the core trial team and practices early in the trial is beneficial for developing relationships. Feedback from one researcher (from the core trial team) was they felt they had a better rapport and a more open channel of communication with those practices in which they attended the site initiation visit. A lot of this required communication between the core trial team and practices related to weekly recruitment updates. At the time, we were using a spreadsheet that was emailed back and forth between researchers and practices. Some practices found this method frustrating, and therefore, if we were to undertake the trial again, we would likely explore the use of a secure web-based platform for the sharing of this information to reduce the burden on the practice research teams.

One event we were unable to predict at the beginning of the PULsE-AI trial was the COVID-19 pandemic and the eventual ‘stay-at-home’ message disseminated by the UK government leading to our trial being paused. During this pause, participants in both intervention and control arms were being diagnosed with AF through routine care, but we were unable to actively recruit or screen any intervention arm participants. On reflection, we could have considered amending the protocol and splitting the intervention arm into two separate cohorts, the first based on all participants who had undergone the screening intervention up to the time at which the ‘stay-at-home’ message was declared, and the other to be a longer-term cohort able to be ‘picked up’ again when research activities were able to resume.

### Limitations

The limitations of the PULsE-AI trial itself have been described in detail elsewhere, [13] however there are potential limitations associated with this reflection exercise. First, while the trial was completed in February 2021, the focus group/interview sessions did not take place until mid-2022. Therefore, is it possible that opinions have changed over time and/or researchers recall of challenges and recommendations is reduced. Second, although approximately half of the members of the TSC participated in the reflection exercise, only one of the six practices involved in the trial accepted the invitation to participate, limiting the diversity of opinions from researchers involved in the trial. The time between trial completion and undertaking this research likely played a role as some of the practice researchers had moved on to new positions at different surgeries and others had moved onto to new research projects and did not have the capacity to participate. We have attempted to prioritise opinions from the participating practice within the analysis, however the poor participation from researchers involved in the trial in this reflection exercise remains a major limitation, limiting the diversity in opinions captured. Last, the nature of the focus group setting may have resulted in researchers voicing fewer contradictory opinions than they may have done in a one-to-one setting. However, the additional value of group sessions is that they can also prompt researchers to share their thoughts on certain topics they may otherwise have not recalled.

## Conclusions

We undertook the PULsE-AI trial to evaluate the effectiveness and cost-effectiveness of a machine learning algorithm (in conjunction with diagnostic testing) for the identification of undiagnosed AF in primary care [14, 27]. The trial was impacted by the COVID-19 pandemic, and following completion, we sought to undertake a reflection exercise to review our own experiences. Given many of our learnings were widely applicable we also wanted to disseminate our learnings with other researchers undertaking or looking to undertake research in the primary care setting. Research is crucial to further our understanding of healthcare and medicine, and as researchers, we need to ensure that we are designing quality trials and providing support to clinicians undertaking research to ensure that these trials are of value, both for furthering knowledge, streamlining pathways and ultimately benefitting patients.

### Electronic supplementary material

Below is the link to the electronic supplementary material.

Supplementary Material 1

## Data Availability

Data that support the findings of this study are available upon reasonable request from the corresponding author.
