# Supplementary material for: Undertaking multi-centre randomised controlled trials in primary care: learnings and recommendations from the PULsE-AI trial researchers
Source: BMC Prim Care. 2024 Jan 2;25:7. doi: 10.1186/s12875-023-02246-8 (PMC10759575; doi:10.1186/s12875-023-02246-8)
Supplement: Supplementary file 1 — Supplementary Material 1 [file 12875_2023_2246_MOESM1_ESM.docx]

**Supplementary file S1.**

**Trial Steering Committee focus group (semi)structure**

**By taking part in this focus group, do you consent that your answers will be used to inform a publication?**

*Recruitment*

1. Across the trial, 28.1% of participants invited to take part in the trial accepted the invitation. How did this response rate compare with your expectations?
   - A. Response rates were lower than I expected
   - B. Response rates were similar to what I expected
   - C. Response rates were higher than I expected
   - D. I am unsure
2. If your answer to question 1. above is A., B., or C., do you have any thoughts as to why response rates were lower than/similar to/higher than expected (or – for those who answered A. or C. – do you think your expectations were unrealistic)?
3. Irrespective of how response rates compared with your expectations, do you have any recommendations on methods to improve response rates in trials in primary care?

*Challenges*

1. What do you think were the key trial-related challenges faced by the trial team and practices during the set-up and running of the PULsE-AI trial?
2. For each challenge, do you think anything could have been done differently (either by the trial team or individual practices) to lessen the impact of these challenges?

*Lessons learned*

1. On reflection, are there any research and/or clinical lessons you’ve learnt from being part of the Trial Steering Committee (TSC) on the PULsE-AI trial? If so, please elaborate.

*Recommendations*

1. Based on your experience, can you think of anything the trial team should do differently to improve the management of the trial if they were to undertake the trial again?

*AI*

1. In your opinion, do you think there is value in the clinical use of artificial intelligence (AI)-based risk equations for patients and clinicians? Please elaborate.
2. As a clinician, would you be willing to employ AI-based risk equations? Please elaborate.
3. If you answered yes to question 9 above, would you consider their outcome alongside your own clinical assessment? Please elaborate.
4. The output of the AF risk prediction algorithm evaluated in the PULsE-AI trial is a continuous variable (percentage) that is then transformed into a binary variable (high vs. low risk) based on a pre-defined threshold. As a clinician, are you willing to trust this binary outcome without knowledge of the continuous output variable? Please elaborate.

*Other*

1. Do you have anything else you would like to share based on your experience in the trial that may be of use to other researchers undertaking trials in primary care?

**Study site interview (semi)structure**

**By taking part in this interview, do you consent that your answers will be used to inform a publication?**

*Recruitment*

1. At your practice, Xx% of participants invited to take part in the trial accepted the invitation. How did this response rate compare with your expectations?
   - A. Response rates were lower than I expected
   - B. Response rates were similar to what I expected
   - C. Response rates were higher than I expected
   - D. I am unsure
2. If your answer to question 1. above is A., B., or C., do you have any thoughts as to why response rates were lower than/similar to/higher than expected (or – for those who answered A. or C. – do you think your expectations were unrealistic)?
3. Irrespective of how response rates compared with your expectations, do you have any recommendations on methods to improve response rates in trials in primary care?

*Challenges*

1. What do you think were the key trial-related challenges faced by your practice during the set-up and running of the PULsE-AI trial?
2. For each challenge, do you think anything could have been done differently (either by yourselves or the wider trial team) to lessen the impact of these challenges?
3. If you were involved in the use of the KardiaMobile/AliveCor technology during the trial, on the below scale, how user-friendly did **you** find the technology? Please choose one of the below:
   - Very easy – easy – neither easy nor challenging – challenging – very challenging
   - If you found it challenging or very challenging, what were the main challenges you encountered?
4. In your opinion, how did **your participants** find the use of the KardiaMobile/AliveCor technology during the trial? Do you recall any direct feedback you can paraphrase?

*Lessons learned*

1. On reflection, are there any research and/or clinical lessons you’ve learnt from taking part in the PULsE-AI trial? If so, please elaborate.

*Recommendations*

1. Based on your experience, is there anything your practice would do differently if you were to undertake the trial again?
2. Based on your experience, can you think of anything the trial team should do differently to improve the management of the trial if they were to undertake the trial again?

*COVID-19*

1. On reflection, how did the pandemic impact your ability to complete the trial?
2. Do you think the pandemic has impacted the detection and management of AF at your practice, and if so, how?

*General*

1. In your opinion, were there any positive aspects relating to your practice’s participation in the PULsE-AI trial? If yes, what were they?
2. Conversely, in your opinion, were there any negative aspects relating to your practice’s participation in the PULsE-AI trial? If yes, what were they?
3. Overall, what is your perception of the benefit vs. the burden of participating in the trial?
4. Do you think your practice’s participation in the trial impacted non-intervention (routine) diagnoses of atrial fibrillation during the trial period? If yes, what was the impact and what do you think was the underlying cause?
5. Do you have anything else you would like to share based on your experience in the trial that may be of use to other researchers undertaking trials in primary care?
